# When Are Sexist Attitudes Risk Factors for Dating Aggression? The Role of Moral Disengagement in Spanish Adolescents

**DOI:** 10.3390/ijerph18041947

**Published:** 2021-02-17

**Authors:** Virginia Sánchez-Jiménez, Noelia Muñoz-Fernández

**Affiliations:** 1 Department of Developmental and Educational Psychology, University of Seville, 41018 Seville, Spain; virsan@us.es; 2 Department of Psychology, Universidad Loyola Andalucía, 41704 Seville, Spain

**Keywords:** sexism, moral disengagement, dating aggression, adolescents, couples

## Abstract

This research aimed to explore the interplay of sexism and moral disengagement (MD) in the explanation of psychological and physical dating aggression. The sample comprised 1113 Spanish adolescents (49.2% girls, *n* = 552) between the ages of 12 to 17 (*M* = 14.44). A latent profile analysis conducted with sub-sample of 432 adolescents with sentimental experience identified four configurations: (1) benevolent; (2) less disengaged and sexist; (3) highly disengaged and sexist; and (4) moderately disengaged and sexist. Regarding gender and age, boys were more present than girls in the moderately disengaged and sexist group, as well as in the highly disengaged and sexist profile. The highly disengaged and sexist and benevolent groups were the youngest. Regarding dating aggression, the highly disengaged and sexist group had the highest engagement in physical and psychological aggression. However, the others three profiles showed a similar engagement in aggression. These findings confirmed the moderating role of MD on the relationship between sexism and dating aggression and suggested that the association between MD, sexism, and dating aggression was exponential; that is, the risk appeared when adolescents were extremely hostile and disengaged. The results have implications for the design of tailored dating aggression prevention programmes.

## 1. Introduction

Dating aggression in adolescent couples is defined as all the aggressive behaviours that occur among members of a romantic relationship or between persons that meet up to go out together. Dating aggression is considered a specific type of intimate partner violence that occurs in first adolescent romantic relationships [1] and is manifested in the form of psychological, physical, sexual, or cyber aggression [2,3]. Studies carried out in recent decades have concluded that aggression in adolescent couples is characterised as moderate [4], contextual or situational [3,5], and bidirectional or reciprocal, at least for psychological and moderated forms of physical aggression [6]. The most common type of violence is that of a psychological nature [7], followed by moderate or mild forms of physical aggression [4], with severe physical and sexual aggression being the least frequent [8,9]. International studies’ prevalence rates have shown a high heterogeneity across studies, with rates between 1% and 61% for physical aggression [2,10], and between 20% and 77% for psychological aggression [10]. Overall, national studies show that Spanish adolescents presented similar rates of psychological dating aggression in comparison to international studies [11], revealing that these psychological tactics are widespread in different societies. However, the rates of physical aggression in Spanish adolescents are higher than in Italian adolescents [6] and North American countries but similar to British adolescents for severe physical aggression [12]. Different authors have pointed out that these higher levels of physical aggression would be an expression of a higher acceptance and normalisation of violence in Spanish adolescents than in other countries. This normalisation could affect their couple quality and satisfaction, as shown in some studies that have found that adolescents involved in dating aggression feel that their current dating relationship is stable and serious [13], scoring high in intimacy and commitment but also exhibiting high levels of conflicts and imbalance of power [6,12]. Ortega-Ruiz and Sánchez-Jiménez [14] have hypothesised that this couple profile reflects a “dirty dating” dynamic, a passive acceptance of being part of a couple where violence and conflicts exist, probably sustained by a strong acceptance of myths of love [15], which could render it difficult to perceive violence and break up the relationship. 

The analysis of risk factors linked to the appearance of these aggressive behaviours within couples revealed the complexity and multidimensionality of the phenomenon [16]. Studies have shown that proximal factors related to the couple context [3,5] are those that seem to play a more significant role in the explanation of dating aggression, specifically the presence of conflicts [17] as well as the partner’s aggressive behaviour [18]. In addition to these factors, sociocognitive variables (e.g., beliefs and attitudes towards gender norms and towards violence) have been associated with dating aggression.

Attitudes towards gender norms, particularly sexism, are social constructions regarding the role of women and men in society. Two forms of sexism have been described [19,20]. Hostile sexism represents the traditional view of women; it is based on beliefs about women’s inferiority and limitations and is expressed by a negative affective tone. Benevolent sexism is considered a new form of sexism. It is expressed through a positive affective tone because it is related to the care and protection of women, although maintaining the restrictive role of women. Studies have found that sexist attitudes are widespread across cultures, men reporting higher levels of hostile sexism than women [21]. In comparison to other countries, Spanish society has experienced fast and significant changes and advances in gender equality in recent decades [22], as represented by the European Institute for Gender Equality (EIGE)’s Gender Equality Index [23], a tool to measure the progress of Gender Equality in Europe. Spain has improved its 2010 gender gaps concerning time spent on care, economic decision-making, and political representation. In this ranking, Spain is in the 8th position, with mean percentages over the European average. However, sexism continues to be present in Spanish adults, more pronounced among men, and it is exemplified in gender roles such as the unequal distribution of household tasks [24]. There is still a large gender gap in this area, despite favourable developments in recent years [23]. Studies on sexism conducted with adolescent population describe the same pattern. Boys present more hostile sexism than girls [25,26,27], but gender differences in benevolent sexism are not conclusive [28]. Longitudinal studies in Spain have shown that sexism increases in the first years of adolescence [27] when boys and girls face pubertal changes, the construction of gender identity and new patterns of interaction with the opposite sex [29]. However, a progressive decreasing trend has been found for benevolent sexism after middle adolescence, but not for hostile sexism [27]. In line with adult samples, the levels of sexism in Spanish adolescents are lower than in other counties for benevolent and hostile sexism [28].

The association between sexism and dating aggression is controversial. Some studies have identified sexism (benevolent and hostile) as a correlate of dating aggression [28,30] and cyber dating aggression [31], whereas others have found this association only for hostile sexism [32,33]. However, other studies have suggested that benevolent sexism could be a protective factor of dating aggression [34]. Furthermore, the direct effect of sexist attitudes on dating aggression is small or moderate [35] which indicates that even when significant, not all the students who present sexist attitudes behave aggressively towards their partners.

According to the multidimensional nature of dating violence, the coexistence of sexist attitudes with other sociocognitive and contextual factors could increase the risk of dating aggression. Previous studies have explored the synergies between sexist attitudes and attitudes towards dating violence in the explanation of dating aggression [36] and victimisation [37]. Reyes and colleagues [36] explored the moderating role of descriptive and injunctive norms about dating violence on the association between gender role beliefs and male dating aggression. In their results, the authors found that gender role attitudes predicted male dating aggression in those who were high in their acceptance of dating violence (injunctive norms) but not in those who were low. Fernández-Antelo and colleagues [37] explored this association in victims. Their results found the interplay between sexism and attitudes towards the abuse as a predictor of victimisation. Specifically, they found that this interaction predicted victimisation only for benevolent sexism, confirming the complexity of dating violence and the need to delve into its nature and predictors in order to prevent the phenomenon from occurring in adolescents. 

Hence, these studies reflect that the impact of sexist attitudes on dating violence is influenced by other factors, such as acceptance of dating violence. However, these attitudes towards dating violence could be considered contextualised factors or domain-specific, directly related to the couple context. In contrast, the influence of the more general process, such as moral disengagement (MD), on the association between sexist attitudes and dating aggression has not been explored to date. In his theory of moral agency, Bandura [38] described a set of cognitive strategies that deactivate individuals’ moral agency, facilitating the individual’s coherence between moral reasoning and moral behaviour. Bandura [38] defined these cognitive tactics as MD mechanisms. Through the deactivation of the behavioural self-regulation process, these cognitive manoeuvres would allow people to buffer the emotional consequences of their violent behaviours, reducing feelings of guilt. Empirical support for the MD theory has been widely demonstrated (see [39,40] for a review), with numerous studies analysing the relationship between MD and aggressive behaviour in adolescents and young people [41,42,43,44] among others. These studies have consistently shown higher levels of MD in boys than in girls [41,45,46] among others and have concluded that MD favours positive attitudes towards violence and bullying [43,47,48]. Regarding dating aggression, there are few studies focused on its association with MD. Rubio-Garay and colleagues [49] found small-to-medium associations between MD and physical and verbal aggression. Cuadrado-Gordillo et al. [50] reported similar results; MD increased the risk for couple victimisation.

In sum, these studies indicate that both MD and sexist attitudes are cognitive correlates of dating aggression, but it is not known how these processes are related to each other in explaining it. To our knowledge, only one recent study has analysed this association in violence against minorities. Carrera-Fernández and colleagues [51] examined the relationship between sexism, homophobia, MD in school bullying and justification of violence against minorities. They found that both forms of sexism were positively related to MD in bullying situations, the association being stronger for hostile sexism than for benevolent sexism. 

### The Present Study

This research aimed to explore the interplay of sexism and MD in the explanation of psychological and physical dating aggression in a sample of Spanish adolescents. In a first step, by using a person-centred approach, this study aimed to identify distinct profiles of adolescents based on their levels of sexism and MD, comparing them in terms of gender and age. In a second step, we analysed whether adolescents belonging to different profiles differed in their involvement in psychological and physical dating aggression.

There is a paucity of studies analysing the association between MD and sexist attitudes as correlates of dating aggression. However, according to previous studies [36,51], we expected that traditional gender roles lead to aggressive behaviour when they are supported by the sociocognitive mechanism that buffers the emotional impact of immoral actions. In contrast, when MD is low, sexist attitudes seem to be insufficient to precipitate aggression and violence towards the partner.

## 2. Materials and Methods

### 2.1. Participants

A total of 1113 adolescents (50.8% boys, *n =* 561; 49.2% girls, *n =* 543; 9 adolescents decided not to provide this information) between the ages of 12 to 17 (*M* = 14.44; *SD* = 1.40) participated in the study. The students were recruited from public secondary schools from Seville and Córdoba (Spain). Concerning their sexual orientation, 1029 adolescents self-identified as heterosexual (95.2%), 12 as homosexual (1.1%), 22 as bisexual (2%), and 18 participants indicated “I don’t know” (1.7%). 

### 2.2. Measures

Moral disengagement was measured using Bandura’s original scale, the Moral Disengagement Scale (MD [52]). Thirty-two items measured on a 5-point Likert scale assessed how much the participants agreed or disagreed with each item, with 0 meaning “I completely disagree” and 4 “I totally agree”. Four items assessed each of the eight MD mechanisms: (1) moral justification (e.g., it is alright to fight to protect your friends); (2) euphemistic language (e.g., slapping and shoving someone is just a joke); (3) advantageous comparison (e.g., damaging some property is no big deal when you consider that others are beating people up); (4) displacement of responsibility (e.g., one kid in a gang should not be blamed for the trouble that the whole gang causes); (5) diffusion of responsibility (e.g., if kids are living under bad conditions they cannot be blamed for behaving aggressively); (6) distortion of consequences (e.g., it is okay to tell small lies because they do not really do any harm); (7) dehumanisation (e.g., some people deserve to be treated like animals); (8) attribution blame (e.g., if kids fight and misbehave in school it is their teacher to blame). 

Dating aggression was measured using the Modified Conflict Tactics Scale (MCTS [53]). In this study we used the Spanish version adapted by Muñoz-Rivas et al. [54]. Two scales of dating aggression were measured. The first scale was psychological aggression and comprised five items measured on a 5-point Likert scale (from 0 = never true to 4 = always true) that assessed several forms of verbal aggression (e.g., insulting or cursing your partner; α = 0.77). The second scale was physical aggression and comprised 9 items measured on a 5-point Likert scale (from 0 = never true to 4 = always true) that assessed several physical acts perpetrated (i.e., pushing, grabbing, or shoving; slapping, kicking, or biting; α = 0.88). Two composite mean scores of psychological and physical aggression were computed by averaging across the items of each subscale.

Hostile and Benevolent sexism was assessed using the Adolescent Sexism Detection (ASD) scale developed by Recio, Cuadrado, and Ramos [20]. Sixteen items measured on a 6-point Likert scale (from 0 = totally disagree to 5 = totally agree) assessed Hostile sexism (e.g., the most suitable place for the woman is her house with her family; α = 0.95). Ten items measured on a 5-point Likert scale (from totally disagree to totally agree) assessed Benevolent sexism (e.g., women are, by nature, more patient and tolerant than men; α = 0.90). Two composite mean scores of hostile and benevolent sexism were computed by averaging across the items of each subscale.

### 2.3. Procedure

This research has the approval of the Research Ethics Committee of the Autonomous Region of Andalucía (code: 1223-N-18). The research team contacted schools based on a list of available schools and informed the heads of schools regarding the research aims and the use of the data for scientific purposes. Once the heads of the schools agreed to participate, families and teachers received information regarding the research project. The questionnaire was administered on paper in a 30 min session by trained researchers during lecture hours. Students could fill in the questionnaire voluntarily after reading the informed consent. Before the questionnaires were completed, trained researchers verbally informed the adolescents of the nature of the study and how their data would be used. Written information was also presented at the beginning of the questionnaire. In case of any doubts, participants could address their questions to the researchers before or during the course of the study. In the case of adolescents under 16 years old, parents’ permission was requested previously by the school staff. The questionnaire was anonymous, and participants did not receive reimbursement. Participants schools received a detailed report with the results obtained. 

### 2.4. Plan of Analysis 

Preliminary analysis: We carried out this analysis with the full sample (*n* = 1113 adolescents). We analysed the factor structure underlying the MD. This decision was justified because of the controversial results obtained in previous Spanish studies which have used the MD scale. In this sense, one study used the original one-dimensional structure without confirming its factorial structure [55]. In contrast, a second study obtained a different solution reorganising the original items into a new classification of cognitive mechanisms [56]. Moreover, international studies have proposed different solutions for the scale, such as eight-factor solutions [44], four-factor solutions [57], and one-dimensional solutions based on 14 items [57,58], 24 items [42], or the original one of 32 items [59]. Hence, three different models (eight-factor, four-factor, and one-dimensional solutions) were tested using Confirmatory Factor Analysis (CFA) (see Table 1). Because the multivariate normality assumption was violated, the MLR estimation method was employed. Models were estimated using full information maximum likelihood (FIML) method for missing values. Standardised factor loadings and correlations among factors were calculated. Furthermore, to assess the models’ overall fit, Chi-squared test statistic, Root Mean Square Error of Approximation (RMSEA), and Comparative Fit Index (CFI) were employed. 

Measurement equivalence of MD across gender and age (early adolescence—from 12 to 14 years old, *n* = 559—and middle adolescence—from 15 to 17 years old, *n* = 554) was also explored using multiple-group analysis. Several steps were taken [62]: configural invariance, metric invariance, and scalar invariance. Evidence of factorial invariance was compared through CFI increase (ΔCFI) between nested models. When the ΔCFI value was above 0.010 [63], full invariance was rejected, suggesting that a model parameter was not invariant. In these cases, partial measurement invariance was calculated considering modification indexes. 

Main analyses: These analyses were performed including only adolescents with sentimental experience (*n* = 432; mean age = 14.35, *SD* = 1.45; 49.1% boys, *n* = 208; 50.9% girls, *n =* 216; 8 adolescents decided not to provide this information). In a first step, descriptive statistics regarding MD, sexism, and dating aggression by gender and age, as well as correlations among MD, sexism, and dating aggression, were estimated. In a second step, we used a person-centred approach to classify students into different profiles of MD, hostile sexism and benevolent sexism. We used a latent profile analysis. Bayesian Information Criterion (BIC), Bootstrapped likelihood ratio test (BLRT), and entropy were considered to decide the number of group profiles. Both lower BIC value and entropy values closer to 1 identified a better solution. When p-value of BLRT is significant, it suggests that the addition of a new profile improves the model fit. Finally, to analyse the discriminant value of these groups on dating aggression, we compared their scores of psychological and physical aggression using ANOVA analysis. The analyses were conducted using MPLUS 8 and SPSS 26.

## 3. Results

### 3.1. Preliminary Results

Results regarding factorial solution of MD are presented in Table 1. Models 1 ^abc^ showed inappropriate standardised correlations (higher than one) among factors and low reliability indexes (between 0.36 to 0.62, except moral justification for the 32-item version that was 0.70). Therefore, these models were not acceptable for Spanish adolescents. 

The models 2 ^abc^ exhibited unacceptable reliability indexes in three dimensions (agency locus, outcome locus, and victim locus) as well as the 32- and 24-item versions displayed CFI values below the cut-off point 0.90 (see Table 1). Therefore, these models were also not acceptable for Spanish adolescents.

Model 3, which integrated eight mechanisms of MD in one dimension, was also tested for the 32-item, 24-item, and 14-item versions. It was observed that the 32- and 24-item versions displayed CFI below the cut-off 0.90 (Table 1), although reliability was excellent (0.90 and 0.89 for the 32- and 24-item versions, respectively). Finally, the 14-item version presented proper adjustment in CFA and excellent reliability (0.83). From this analysis of the theoretical and empirical models of MD, it was concluded that the one-dimensional model (14-item version) seemed to show the best psychometric properties, in terms of reliability and construct validity.

In a second step, multiple-group analysis of MD (one dimensional, 14-item version) by gender and age was carried out. Results supported partial scalar invariance across gender and age (Table 2). This factorial solution was used in the following analysis.

### 3.2. Main Results

Descriptive analysis of MD, dating aggression, and sexism is described in Table 3. Results showed that boys displayed higher levels of MD and hostile sexism than girls. In contrast, girls exhibited higher levels of psychological aggression than boys. For the comparison between early (12–14 years old) and middle (15–17 years old) adolescents, middle adolescents presented higher levels of psychological aggression than early adolescents. In contrast, early adolescents supported more frequently hostile and benevolent sexism than middle adolescents. 

Correlation analysis indicated that MD was related to hostile sexism (r = 0.46, *p* < 0.001), benevolent sexism (r = 0.41, *p* < 0.001), psychological aggression (r = 0.24, *p* < 0.001), and physical aggression (r = 0.32, *p* < 0.001). Hostile sexism was associated with benevolent sexism (r = 0.70, *p* < 0.001), psychological aggression (r = 0.14; *p* < 0.001), and physical aggression (r = 0.25, *p* < 0.001). Similarly, benevolent sexism was positively related to dating aggression (r = 0.19, *p* < 0.001) for both psychological and physical aggression. 

Latent profile analysis (LPA) was carried out to explore distinct profiles of MD and sexism among adolescents. Fit indices for solutions from two to four latent profiles are displayed in Table 4. Considering BLRT, all models showed significant p-values, which suggests that the addition of one more profile led to an improvement in the model fit. BIC values were similar among the two to four solutions, and entropy was equal to or above 0.80 for all solutions; for this reason, we decided that the best model was the four-profile solution. This solution can classify groups with distinct patterns of MD and sexism. 

**Figure 1:**
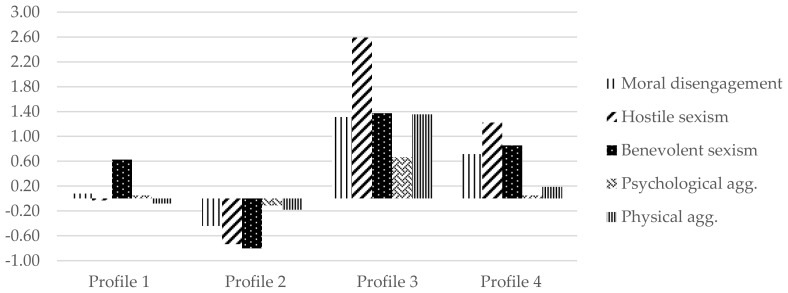
Four-profile solution based on the estimated z-scores for MD, hostile and benevolent sexism, and psychological and physical dating aggression. Profile 1 = benevolent; Profile 2 = less disengaged and sexist; Profile 3 = highly disengaged and sexist; Profile 4 = moderately disengaged and sexist.

The structure of profiles with raw scores is shown in Table 5, and z-scores are depicted in Figure 1. In the first profile, 23.5% (*n* = 96) of adolescents presented moderate scores of benevolent sexism but low MD and hostile sexism (labelled benevolent). The second profile was the most frequent (51.7%; *n* = 211). In this second profile, adolescents reported low levels of MD and sexism (benevolent and hostile). We labelled this profile as the less disengaged and sexist group. In the third profile, 5.9% (*n* = 24), participants were characterised by presenting very high levels of hostile sexism, together with high levels of benevolent sexism and MD. This profile was named highly disengaged and sexist. Finally, the fourth group, 18.9% (*n* = 77), included adolescents who presented moderate levels of MD and moderate benevolent and hostile sexism. We named this group moderately disengaged and sexist. 

Gender was distributed unequally among the distinct profiles, X^2^(3) = 22.82, *p* < 0.001. In the benevolent and less disengaged and sexist groups, a higher proportion of girls than boys were classified (13.9% girls vs. 8.7% boys, and 28.1% girls vs. 24.1% boys, respectively). In contrast, in highly disengaged and sexist and moderately disengaged and sexist groups, boys were more present than girls were (5% boys vs. 0.8% girls, and 12.3% boys vs. 7.1% girls, respectively). Regarding age (see Table 5), the profile of less disengaged and sexist adolescents was the oldest. In contrast, the groups highly disengaged and sexist and benevolent were the youngest. 

Finally, it was investigated whether adolescents classified in these distinct profiles displayed differences in terms of dating aggression (psychological and physical). Results suggested statistical differences between the groups (see Table 5). Post-hoc analyses described that the profile of highly disengaged and sexist adolescents presented higher levels of physical and psychological aggression in comparison to the other three profiles. However, no differences in dating aggression were found among benevolent, less disengaged and sexist, and moderately disengaged and sexist groups. 

## 4. Discussion

The aim of this study was to deepen the understanding of the associations between MD, sexism, and dating aggression in adolescents. To do so, a latent profile analysis was performed to group adolescents by their levels of MD and sexism. These profiles were then compared in terms of physical and psychological dating aggression. 

Four profiles of adolescents were found. The largest group, representing 50% of the adolescents, showed low levels of benevolent and hostile sexism as well as low levels of MD. Around a quarter of adolescents presented moderate levels of benevolent sexism but low levels of hostile sexism and MD. Almost 20% of the adolescents showed moderate levels of sexism (benevolent and hostile) and MD. The fourth group was the least numerous and was characterised by high levels of sexism and MD. In general terms, the profiles obtained provided evidence for the association between MD and both forms of sexism but indicated a stronger association between hostile sexism and MD, as a previous work in the area has concluded [51]. In our study, the benevolent group consisted of a considerable percentage of adolescents who showed moderate levels of benevolent sexism but low levels of hostile sexism and MD. However, an increase in hostile sexism was accompanied by high levels of MD (and vice versa) and high levels of benevolent sexism, as reflected by the profiles highly disengaged and sexist, and moderately disengaged and sexist. In sum, these profiles suggest that those adolescents who exhibit high levels of traditional and hostile gender attitudes tend to use MD to justify transgressive behaviours and minimise the seriousness of dating violence [64]. However, the acceptance of conventional gender roles, based on the care and protection of women that characterised benevolent sexism, was not necessarily accompanied by MD and hostile attitudes, at least not for all the adolescents.

Gender and age differences were found in the different profiles. Boys and younger adolescents were overrepresented in the groups moderately disengaged and sexist and highly disengaged and sexist, showing average and high levels of hostile and benevolent sexism and MD. In contrast, girls were overrepresented in the group less disengaged and sexist, presenting low levels of sexism and MD. These results are similar to those of prior studies that have found that boys scored high in MD and sexism [25,45,46] and that the acceptance of sexism decreases with age [65,66]. In terms of intervention, these results indicate how important it is to address these issues at early ages to prevent the consolidation of dominant and aggressive dynamics in the first romantic relationship [16]. 

These profiles were also different in their involvement in dating aggression. Specifically, those adolescents classified in the highly disengaged and sexist group presented higher levels of psychological and physical aggression. In contrast, participants of the other groups (benevolent, less disengaged and sexist, and moderately disengaged and sexist) did not show differences in their rates of dating aggression. These results are relevant because they expand the understanding of the association between sexism and dating aggression in a context where previous studies have shown controversial results. For instance, some works have found that both hostile and benevolent sexism are directly related to dating aggression [28,31,67]. Others have pointed to hostile sexism as directly correlated to aggression [33,68,69], yet other authors have highlighted that benevolent sexism could be a risk factor for victimisation [37] but a protective factor against aggression [34]. Our results seem to be in line with those studies that emphasised the contribution of both hostile and benevolent sexism to dating aggression, but also indicate that only high levels of sexism and mostly very high levels of hostile sexism put adolescents at risk of dating aggression. These adolescents also presented high levels of MD, facilitating the justification of their actions and minimising emotional discomfort when aggression towards the partner occurred [49].

Hence, the findings of this study confirm our previous hypothesis regarding the shaping role of MD in the relationship between sexism and dating aggression. As our results showed, dating aggression was not present in the romantic relationships of those adolescents who displayed moderate levels of benevolent sexism and low MD (benevolent group), and neither was it evident for those adolescents belonging to the moderately disengaged and sexist group. On the contrary, only when the adolescents manifested high levels of sexism (hostile and benevolent) accompanied by high levels of MD did the rates of aggression in the couple escalate. According to Moral Disengagement Theory [38], our results would suggest that adolescents with high levels of sexism could endorse interaction schemes in the context of romantic relationships based on dominance toward girls. However, the translation of these scripts into specific aggressive behaviours would need sociocognitive mechanisms to justify the use of violence to express dominance. Although our study has a cross-sectional design, previous longitudinal research has found that injunctive normative beliefs moderated the association between gender roles and dating aggression in boys [36]. Future studies would advance this result, which requires further, more in-depth analysis. Moreover, our results suggested that the association between MD, sexism, and dating aggression was exponential; dating aggression did not progressively increase in parallel to sexism and MD. The risk appeared only when adolescents were extremely hostile and disengaged. Overall, these findings allow us to understand the low-to-moderate correlations between dating aggression and sexism [35] and MD [49] that have been found in studies focused on the direct association of sexism and MD and dating aggression. According to the multidimensional nature of dating violence, it is important to understand how individual and contextual risk factors interact to precipitate aggression. In comparison to these previous studies, our work used a more comprehensive approach that takes into account the joint contribution of different variables in the explanation of dating aggression. This profile analysis also considered the gender and age effect, concluding that the high-risk group (highly disengaged and sexist) was composed mainly of younger boys. Identifying this target group and its risk of dating aggression in comparison to the other three groups is extremely relevant for the design of universal and tailored interventions. In agreement with our results, certain levels of sexism and MD could be expected in community samples, but these levels were not related to increased dating aggression, as reflected in the profiles Less disengaged and sexist, benevolent, and moderately disengaged and sexist. These results would indicate that risk factors, other than MD and sexism factors, influence dating aggression. Consequently, universal interventions aimed at reducing dating violence, at least those of short duration, should include not only the content of sexism or acceptance of violence beliefs. In other words, these interventions could change sexist attitudes and beliefs but not physical and psychological dating aggression, in line with the results of meta-analysis and systematic reviews of the efficacy of dating violence prevention programmes [70,71]. In contrast, for the design of selective interventions, this study describes a profile group composed of young boys who need intensive work to change their sexist attitudes and sociomoral mechanisms to reduce their levels of physical and psychological dating aggression. The development of selective and indicated interventions continues to be a challenge for dating violence intervention research because its design needs an accurate analysis of the group needs [72]. However, the efficacy of these selective interventions is showing promising results [73,74,75,76]. This study offers some insights for the development of tailored interventions in risk groups according to the level of MD and sexism they present.

This study also explored the dimensionality of the MD scale among Spanish adolescents. The confirmatory factor analyses and reliability indexes confirm that the MD scale can be used as a one-dimensional structure for Spanish adolescents. These data are in line with those reported in the literature. Similarly, there is current empirical evidence that confirms both one-dimensional structure [62,63,77,78,79] and four-dimensional structure [57,80]. In the present study, the reliability indexes calculated for the four-dimensional structure showed a lack of consistency in three of the four dimensions, discouraging the use of this solution for Spanish adolescents. Future studies could include new items in the three dimensions where reliability indexes were unacceptable to improve the consistency of these subscales, in line with the Australian adaptation of the scale [80]. 

Despite the strength of this study, some limitations have been identified. The sample size employed in the latent profile analysis was small, calling for the replication of this study using larger samples. Given the cross-sectional nature of the study, no temporal association can be established between MD, dating aggression, and sexism. The study variables were measured by self-report questionnaires so that the response bias could be present in the study results. Future research adopting a longitudinal design could corroborate the relationship between the variables and more deeply explore the role of MD on the association between sexism and dating aggression. 

## 5. Conclusions

The aim of this study was to deepen the understanding of the association between MD, sexism, and dating aggression in adolescent couples. Using a person-centred approach, we identified four profile groups that differed in terms of their level of MD and sexism. These profiles showed different levels of involvement in dating aggression. Specifically, those adolescents who presented high levels of sexism (benevolent and hostile) and MD were those more engaged in physical and psychological aggression. Moreover, these adolescents were the youngest and mainly boys. The other profiles were not related to dating aggression. These results contribute to knowledge regarding the interplay between MD and sexism in the explanation of dating aggression. Furthermore, these findings provide guidance for the design of psychoeducational programmes to prevent dating aggression in adolescence at the universal and selective level.

## Figures and Tables

**Table 1:** Fit indices of the models for moral disengagement (MD).

Models	X^2^	df	RMSEA	CFI
Model 1 ^a^ (8 factors and 32 items)	1244.36	436	0.04	0.86
Model 1 ^b^ (8 factors and 24 items)	663.31	224	0.04	0.90
Model 1 ^c^ (8 factors and 14 items)	150.78	56	0.04	0.95
Model 2 ^a^ (4 factors and 32 items)	1525.03	458	0.05	0.82
Model 2 ^b^ (4 factors and 24 items)	923.64	246	0.05	0.84
Model 2 ^c^ (4 factors and 14 items)	234.74	71	0.05	0.92
Model 3 ^a^ (1 factor and 32 items)	1684.16	464	0.05	0.79
Model 3 ^b^ (1 factor and 24 items)	984.71	252	0.05	0.84
Model 3 ^c^ (1 factor and 14 items)	253.09	77	0.05	0.92

Note: Model 1 = original validation of eight factors proposed by Caprara and colleagues [59]. Model 2 = theoretical classification of eight mechanisms of MD in four dimensions (second order factor) [59]; Model 3 = one-dimensional validation of the scale MD [60,61]; ^a^ 32 items version [59]; ^b^ 24 items for middle school [59]; ^c^ 14 items for elementary school [59]. Abbreviations: X^2^, Chi-square test statistic; df, Degree of Freedom; RMSEA, Root Mean Square Error of Approximation; CFI, Comparative Fit Index.

**Table 2:** Multi-group analysis for MD (1 factor and 14 items) across gender and age.

Models	X^2^	df	RMSEA	CFI	ΔCFI
CFA girls ^a^	146.38	74	0.043	0.906	
CFA boys	168.80	77	0.047	0.922	
Multiple-group analysis across gender					
Configural	165.58	151	0.045	0.916	
Metric	347.78	164	0.045	0.907	−0.009
Scalar	419.57	177	0.051	0.875	−0.032
Scalar partial (relaxing intercept item 9)	387.22	176	0.047	0.891	−0.016
Scalar partial (relaxing intercepts item 9 and item 32)	368.15	175	0.045	0.900	−0.007
CFA early adolescence	132.03	77	0.037	0.942	
CFA middle adolescence ^b^	177.24	75	0.050	0.912	
Multiple-group analysis across age					
Configural	312.07	152	0.044	0.924	
Metric	322.42	165	0.042	0.925	0.001
Scalar	368.51	178	0.044	0.910	−0.015
Scalar partial (relaxing intercept item 26)	355.67	177	0.043	0.915	−0.010
Scalar partial (relaxing intercept item 26 and item 32)	348.03	176	0.043	0.918	−0.007

Note: ^a^ Three covariance errors were added (item 24 with item 31, item 22 with item 26, and item 19 with item 3); ^b^ two covariance errors were added (item 24 with item 22; item 31 with item 23). Abbreviations: CFA, Confirmatory Factor Analysis; X^2^, Chi-square test statistic; df, Degree of Freedom; RMSEA, Root Mean Square Error of Approximation; CFI, Comparative Fit Index; ΔCFI, increment of Comparative Fit Index between the nested models.

**Table 3:** Descriptive analysis by gender and age.

Study Variables		Gender			Age		
	M (SD)	Boys	Girls	T-Value (df); Cohen’s d	Early Adolescents	Middle Adolescents	T-Value (df); Cohen’s d
MD	0.90 (0.78)	1.07 (0.86)	0.73 (0.63)	t (394) = 4.420 **; d = 0.45	0.88 (0.75)	0.91 (0.80)	t (402) = −0.428; d = −0.04
Hostile sexism	1.05 (1.19)	1.33 (1.37)	0.80 (0.94)	t (357) = 4.343 **; d = 0.46	1.24 (1.23)	0.85 (1.11)	t (364) = 3.197 **; d = 0.34
Benevolent sexism	1.82 (1.32)	1.84 (1.32)	1.83 (1.31)	t (360) = 0.056; d = 0.01	2.08 (1.30)	1.56 (1.29)	t (367) = 3.803 **; d = 0.40
Psychological aggression	1.07 (0.93)	0.80 (0.89)	0.97 (0.79)	t (385) = −2.046 *; d = 0.21	0.75 (0.84)	1.03 (0.81)	t (393) = −3.359 **; d = 0.34
Physical aggression	0.18 (0.47)	0.16 (0.47)	0.18 (0.46)	t (390) = −0.389; d = 0.04	0.15 (0.41)	0.20 (0.52)	t (398) = −1.101; d = 0.11

Note: Mean values (standard deviation) are displayed in the table; ** *p* ≤ 0.01; * *p* ≤ 0.05. Abbreviations: M, Mean; SD, Standard Deviation; df, Degree of Freedom

**Table 4:** Fit indices for the latent profile analysis (LPA) solutions.

# of Profiles	BIC	BLRT	Entropy
2	3100.46	371.02 ***	0.86
3	2984.65	139.85 ***	0.81
4	2895.91	113.58 ***	0.84

Note: *** *p* < 0.001. In the five-profile solution, the standard error of hostile sexism was 0 in class 3, and only two participants were included in this class. For these reasons, this solution was not considered. Abbreviations: #, number; BIC, Bayesian Information Criterion; BLRT, Bootstrapped likelihood ratio test.

**Table 5:** Structure of profiles, age, and dating aggression differences among the four identified MD and sexism profiles.

Title	Profiles					
	Benevolent	Less Disengaged and Sexist	Highly Disengaged and Sexist	Moderately Disengaged and Sexist	*F*-Value	d
Structure						
MD	0.99 (0.58) ^a^	0.57 (0.51) ^b^	1.95 (1.31) ^c^	1.41 (0.81) ^d^	51.24 ***	0.46
Hostile sexism	1.02 (0.37) ^a^	0.18 (0.23) ^b^	4.07 (0.61) ^c^	2.50 (0.41) ^d^	1358.52 ***	1.80
Benevolent sexism	2.65 (0.84) ^a^	0.74 (0.64) ^b^	3.58 (1.39) ^c^	2.99 (0.67) ^d^	246.44 ***	1.16
Distribution						
Age	14.20 (1.34) ^a^	14.77 (1.50) ^b^	14.21 (1.65) ^a^	14.48 (1.46) ^b^	5.873 ***	0.26
Dating aggression						
Psychological agg.	0.86 (0.72) ^a^	0.80 (0.73) ^a^	1.58 (1.54) ^b^	0.96 (0.86) ^a^	6.149 ***	0.19
Physical agg.	0.13 (0.28) ^a^	0.11 (0.38) ^a^	0.78 (1.22) ^b^	0.26 (0.55) ^a^	13.768 ***	0.21

Note: Mean values (standard deviation) are displayed in the table; a–d: Means with a different superscript differed significantly. *** *p* < 0.001.

## Data Availability

The datasets generated and analyzed during the current study are available from the corresponding author on reasonable request.
